# Observation of Chinese Hamster Ovary Cells retained inside the non-woven fiber matrix of the CellTank bioreactor

**DOI:** 10.1016/j.dib.2015.10.006

**Published:** 2015-10-20

**Authors:** Ye Zhang, Véronique Chotteau

**Affiliations:** School of Biotechnology, Department Industrial Biotechnology/Bioprocess Design, Cell Technology Group (CETEG), Royal Institute of Technology, KTH, SE-10691 Stockholm, Sweden

## Abstract

This data article shows how the recombinant Chinese Hamster Ovary (CHO) cells are located in the interstices of the matrix fibers of a CellTank bioreactor after completion of a perfusion culture, supporting the article entitled “Very high cell density perfusion of CHO cells anchored in a non-woven matrix-based bioreactor” by Zhang et al. [1]. It provides a visualization of the cell distribution in the non-woven fiber matrix in a deeper view.

**Specifications table**

**Table:** 

Subject area	Biology
More specific subject area	Microscopy
Type of data	Movie of Z-stack image sequence
How data was acquired	Microscope, Leica DMI6000
Data format	Raw
Experimental factors	Non-woven fiber scaffold was sliced with a sharp blade and a fluorescent dye DAPI staining cell nuclei was used to visualize the cell distribution inside the scaffold
Experimental features	The cell nuclei distribution was examined along the *Z*-axis
Data source location	KTH, Stockholm, Sweden
Data accessibility	Data is with this article

**Value of the data**

• The movie clip helps to visualize the distribution of the cells inside the matrix in a deeper view.
• The data helps to understand the concept used in the CellTank. In this bioreactor, the cells are retained in the interstices of the non-woven fiber matrix.
• The data supports that the cells, which are normally cultured in suspension (i.e. non-adherently), are entrapped in the interstices between the fibers of the matrix instead of adhering to the fibers.
• There are similar technologies for cell entrapment or anchorage during cultivation, for example, depth-filter bioreactor, fibrous bed bioreactors, packed beds with carriers disks of type Fibra-cell, electrospun fiber scaffolds, where the present visualization technique can be used. The data sheds light on distribution of the cells inside such fiber-based scaffolds.

## 1 Data

The data composed of 56 pictures in 85,90 µm depth along the *Z*-axis, shows the fiber matrix and cell nuclei stained with Dapi at cell density 152×10^6^ cells/mL after 34 days perfusion cultivation, taken by a fluorescence microscope.

## 2 Experimental design, materials and methods

The CellTank is a perfusion-integrated Single-Use-Bioreactor (SUB), see Supplementary material 1, for a sketch of the bioreactor design. A 150 cm^3^ cassette containing polyester non-woven spun fiber matrix, used for the cell retention, is immersed in a 2 L reservoir where the perfusion of the culture medium takes place. During the culture in this system, it is not possible to take cell samples from the matrix due to the fact that the cells are entrapped in the fiber matrix. This is the reason why the cell density has been measured with an on-line biomass sensor [1]. To better understand how the cells are sitting inside the fiber matrix, the bioreactor and the cassette have been disassembled at the end of a cultivation run and the matrix scaffold has been examined by fluorescence microscopy.

### 2.1 Sample sectioning

At the end of a 34-day perfusion cultivation, the final biomass reading from the on-line biomass probe was 152×10^6^ cells/mL. The bioreactor was then disassembled and 10 fiber disks were individually removed from the cassette [1]. The disks were placed in petri dishes, and a sharp blade was used to manually slice the 5 mm thick disks by orthogonal cuts into thin strips to obtain a section view through the disk.

### 2.2 Fluorescence microscopy

It was impossible to perform the microscopy of the fiber disk under bright field due to the fact that the cells could not been distinguished from the fibers in this light. Using fluorescence microscopy, the cell nuclei were easily visualized and the fibers were less interfering due to a better contrast brought by the DAPI staining. Prior microscopy analysis, the sample strips were fixed with 4% paraformaldehyde for 20 min, washed three times with PBS, placed on a microscope slide and 3 drops of histology mounting medium Fluoroshield™ with DAPI (F6057, Sigma) were put on top of the sample. The cell distribution was then observed by fluorescence microscopy up to a depth of 300 µm (inverted microscope Leica DMI6000b). A short movie clip was taken by stacking 56 pictures with 85,90 µm depth along the *Z*-axis.

## Footnotes

Appendix A Supplementary data associated with this article can be found in the online version at doi:10.1016/j.dib.2015.10.006.
