# Supplementary material for: Observation of Chinese Hamster Ovary Cells retained inside the non-woven fiber matrix of the CellTank bioreactor
Source: Data Brief. 2015 Oct 20;5:586–8. doi: 10.1016/j.dib.2015.10.006 (PMC4773380; doi:10.1016/j.dib.2015.10.006)
Supplement: Supplementary file 1 — Supplementary material [file mmc1.zip › Supplementary material 1.pdf]

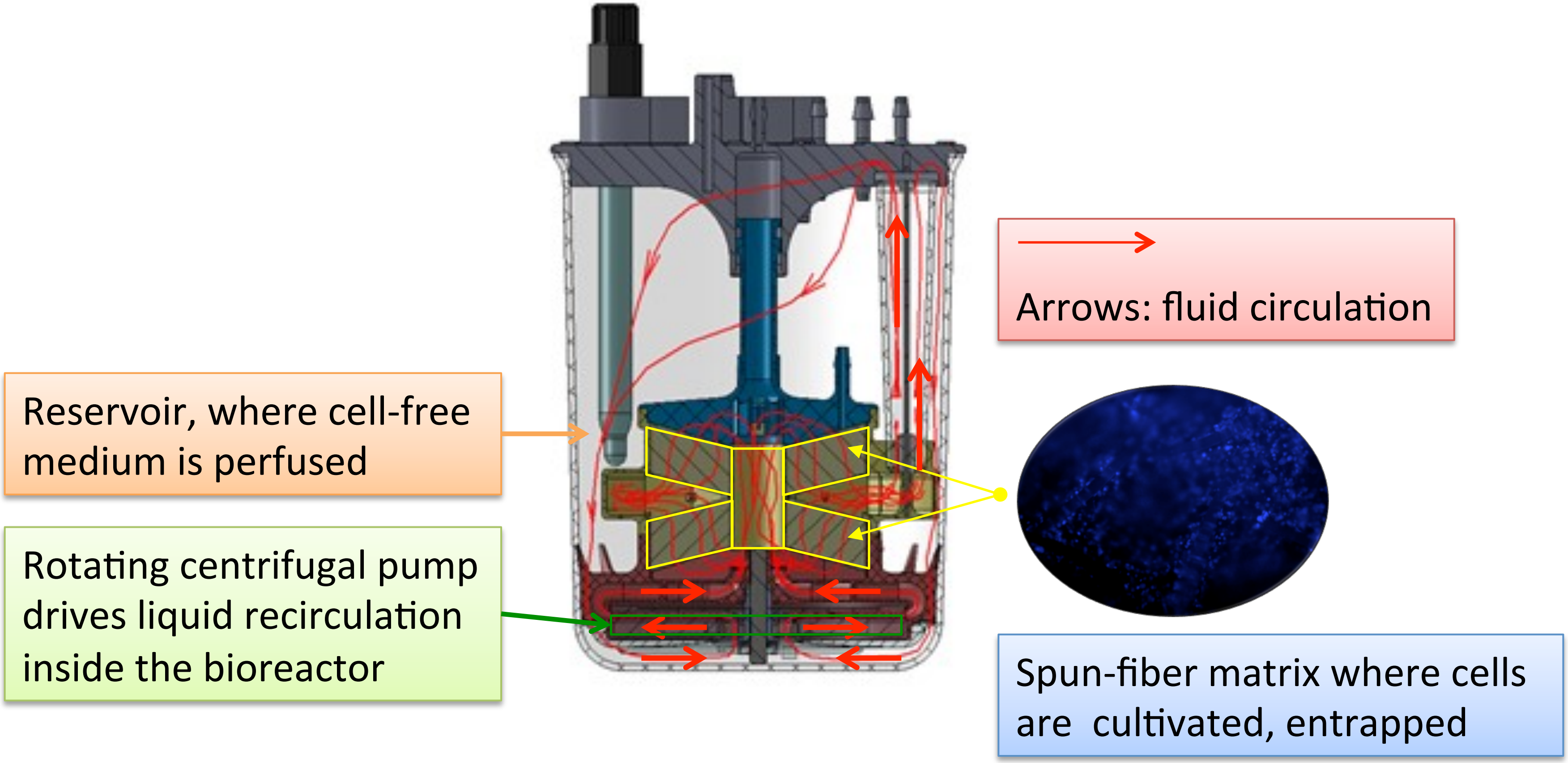
Arrows: fluid circulation
Reservoir, where cell-free medium is perfused
Rotating centrifugal pump drives liquid recirculation inside the bioreactor
Spun-fiber matrix where cells are cultivated, entrapped
