# Supplementary figures and images for: Observation of Chinese Hamster Ovary Cells retained inside the non-woven fiber matrix of the CellTank bioreactor
Source: Data Brief. 2015 Oct 20;5:586–8. doi: 10.1016/j.dib.2015.10.006 (PMC4773380; doi:10.1016/j.dib.2015.10.006)

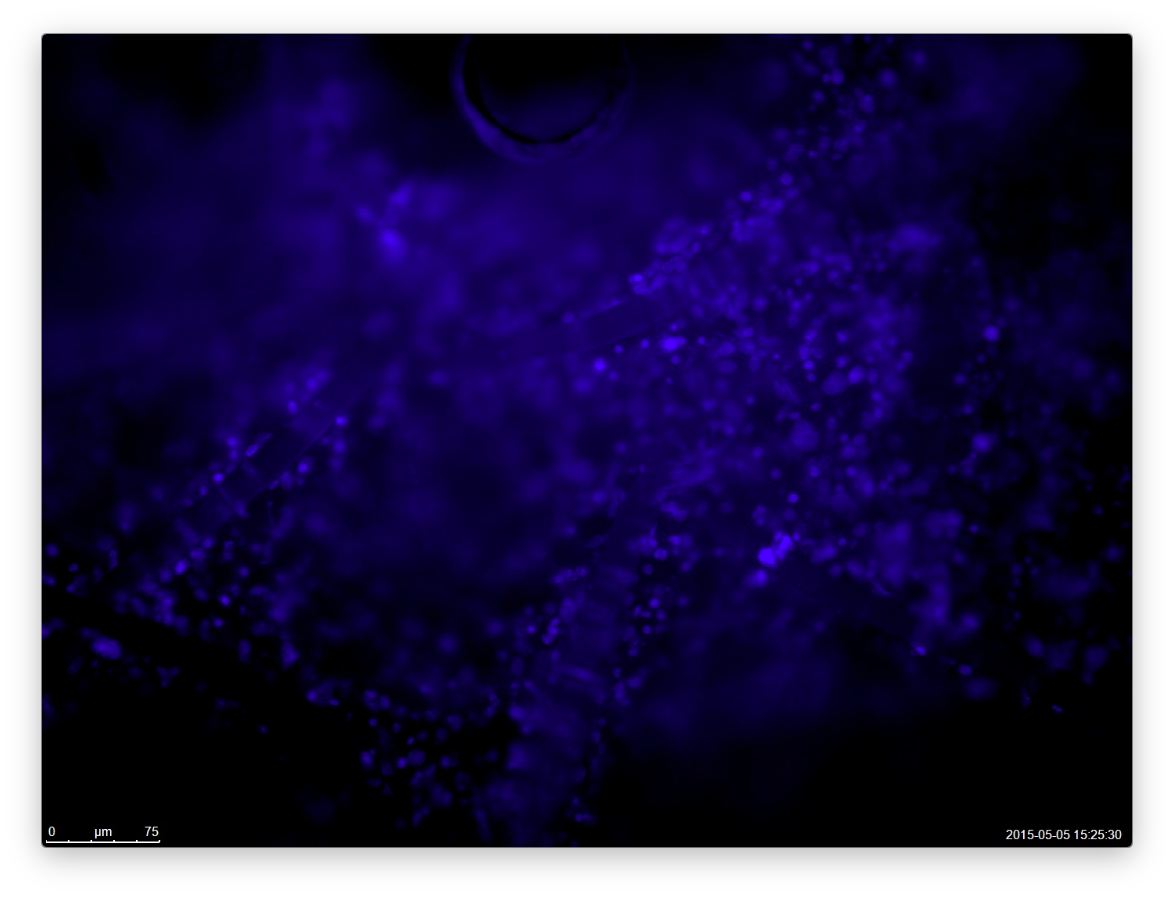

Supplement: Supplementary file 1 — Supplementary material [file mmc1.zip › thumbnail for the data movie.png]
